# Nurses’ ethical challenges caring for people with COVID-19: A qualitative study

**DOI:** 10.1177/0969733020944453

**Published:** 2020-08-28

**Authors:** Yuxiu Jia, Ou Chen, Zhiying Xiao, Juan Xiao, Junping Bian, Hongying Jia

**Affiliations:** 531675 The Second Hospital of Shandong University, China; 531675 Shandong University, China; 531675 The Second Hospital of Shandong University, China

**Keywords:** COVID-19, ethical challenges, nurses, qualitative research

## Abstract

**Background:**

Ethical challenges are common in clinical nursing practice, and an infectious environment could put nurses under ethical challenges more easily, which may cause nurses to submit to negative emotions and psychological pressure, damaging their mental health.

**Purpose:**

To examine the ethical challenges encountered by nurses caring for patients with the novel coronavirus pneumonia (COVID-19) and to provide nurses with suggestions and support regarding promotion of their mental health.

**Research design and method:**

A qualitative study was carried out using a qualitative content analysis. The participants were 18 nurses who agreed to attend an interview and describe their own experiences of providing care to COVID-19 patients in China. They were purposively sampled, and structured, in-depth interviews were performed. Data were iteratively collected and analyzed from February to March 2020.

**Ethical considerations:**

The proposal was approved by the Research Ethics Committee of the Second Hospital of Shandong University, China.

**Findings:**

The findings revealed three main themes and 10 categories. The themes were the following: (1) ethical challenges (people with COVID-19, inequality, professional ethics, and job competency); (2) coping styles (active control and planning, seeking support as well as catharsis, and staying focused); and (3) impacts on career (specialized nursing skills, scientific research ability, and management skills).

**Conclusion:**

Nurses faced ethical challenges on multiple fronts in caring for COVID-19 patients. The results may help nurses with more safety, ethics, and humanistic care in nursing practice.

## Introduction

Ethical challenges are common in clinical nursing practice.^1–3^ The expression “ethical challenges” mainly refers to ethical dilemmas and ethical conflicts as well as other scenarios where difficult choices have to be made.^4^ Ethical dilemmas are described as situations that cannot be solved; decisions made between two options may be morally plausible, but are equally problematic due to the circumstances.^5^ Ethical conflicts, on the contrary, arise when one is aware of the necessity of proper actions but he or she may have trouble exercising these actions because of certain internal or external factors.^6^ Studies have shown that nurses are likely to face ethical challenges on multiple fronts in clinical practice.^7^

Novel coronavirus pneumonia, also known as COVID-19, is a type of pulmonary inflammation caused by novel coronavirus infection. It has been spreading worldwide since December 2019. The virus can be transmitted between humans,^8^ and almost everyone is susceptible to COVID-19.^9^ Around one out of every five people who get COVID-19 becomes seriously ill and develops difficulty in breathing.^10^

The novel coronavirus is mainly transmitted through droplets, close contact,^11^ or air through aerosols generated by medical operations,^12^ and therefore, medical personnel treating people with COVID-19 are more likely to be exposed to COVID-19 environment. When nurses are treating people with contagious diseases, the ethical challenges posed by treating infectious diseases may include fear of infection, disappointing results of treatment, and high mortality rate.

As the primary caregiver of people with COVID-19, nurses accounted for 68% of the medical workers in China.^12^ To contain the disease outbreak, new wards were established to concentrate patients. Nurses caring for COVID-19 patients faced difficulties such as an unfamiliar working environment, exposure to the disease, lack of experience in their new jobs, and close attention from the general public and media. These elements can put nurses under ethical challenges,^13^ which may cause nurses to submit to negative emotions (such as anxiety or fear) and psychological pressure (such as insomnia or irritability), damaging their mental health.^14^ A qualitative approach may offer important insight into the ethical challenges experienced by nurses caring for COVID-19-positive patient populations in China.

## Purpose

The purpose of the study was to examine the ethical challenges encountered by nurses caring for patients with COVID-19 and share their coping styles to ethical conflicts and dilemmas. It was anticipated that results might provide nurses with suggestions and support regarding promotion of their mental health.

## Materials and methods

### Design

A descriptive qualitative study was taken to examine the ethical challenges of nurses treating COVID-19 patients. As a research method for making replicable and valid inferences from data, content analysis was considered.

### Participants

Nurses were purposively sampled from those who work on designated units to take care of COVID-19 patients in Wuhan from Shandong Province. By contacting the leader of these nurses, the participants were recruited. We then contacted the potential participants provided by the leader by phone. If the individuals were willing to attend an interview and to describe their own experiences, they were asked to complete a written consent form. We had a pre-interview with four experienced nurses, which made us master interview skills and also made the interview outline more reasonable. Then, the subsequent sampling was taken. Sampling ceased when data saturation (no new information emerging) was reached. In order to enrich the diversity of data, 18 nurses of different ages, genders, and specialties were selected, whose information is given in Table 1.

**Table 1.** Demographic characteristics of the participants.

Characteristics	*n*
Gender	
Female	13
Male	5
Age (years)	
20–29	7
30–39	7
40–49	4
Education level	
Bachelor	14
College diploma	4
Work experience (years)	
<5	3
5–9	6
10–19	6
≥ 20	3
Profession	
Outpatient	2
Internal medicine	8
Surgery	3
Emergency	2
ICU	3

ICU: intensive care unit.

### Data collection

Data were collected through structured in-depth interviews from February to March 2020. Based on the International Council of Nurses’ (ICN) Code of Ethics for Nurses,^15^ the preliminary interview outline was drafted with consulting doctors and nurses providing frontline care for people with COVID-19 and with experts who specialize in contagious diseases, nursing management, and nursing education. According to the four pre-interview results, the final interview outline was created. The main questions were the following:

1. What ethical challenges did you encounter in nursing COVID-19 patients?
2. How did you cope with these ethical challenges?
3. What impact do you think these challenges will have on your career?

First, researchers explained to the participants about the definition and forms of ethical challenges as well as the method of qualitative interviewing. Then, considering the contagious nature of COVID-19,^11^ all sessions were held in the form of a video interview or voice chat based on participants’ wishes.^16^ Two main researchers conducted all interviews at a pre-arranged time. In this study, 20 interviews were conducted, including 4 pre-interviews, 14 formal interviews, and 2 secondary interviews. Each interview took 60–120 min, and the average time was 92 min. With the permission of the participants, the statements were recorded and transcribed verbatim at the end of each interview.

### Data analysis

Within 24 h after each interview, the recorded materials were transcribed word-by-word into written materials, with non-verbal information included. The research data were analyzed using the content analysis method.^17^ Researchers read the written materials several times and noticed latent content, such as silence, laughing, sobbing, and posture. Inductive content analysis was used to organize the data, including coding, creating categories, and abstraction. To ensure the reliability of the research results, data analysis was carried out by two researchers at the same time.^18^ If a controversial theme emerged, they would consult with the third researcher. The process was followed by an experienced qualitative researcher.

### Rigor

Pre-interviews were conducted before the formal interviews to ensure the rationality of the interview outline and the representativeness of the objects. The transcribed data and non-verbal information were analyzed repeatedly by two researchers to increase the reliability. The researchers provided rich description of the research context, selection of participants, and data collection and analysis process to facilitate transferability of this study.^19^

### Ethical consideration

This study was approved by the Research Ethics Committee of the Second Hospital of Shandong University (Ethical code: KYLL-2020(LW)-023.). Potential participants were informed of the aims and purpose of the study and were provided with a Participant Information Sheet, which was conducted by a professional researcher via chat tools or e-mail. If voluntary consent was given, participants were invited to sign the consent form prior to participation in the study.

### Findings

A total of 18 eligible nurses were recruited, who were aged 24–43 years and had a nursing tenure of 3–22 years. They shared ethical challenges of caring for COVID-19-positive patients and also their coping styles. Based on the descriptions of nurses, three themes and 10 categories with 8 subcategories were revealed, which are provided in Table 2.

**Table 2.** Summary of themes and subthemes.

Themes	Main category	Subcategory
Ethical challenges	People with COVID-19	Neglected patient rights
		The lack of emotional support
	Inequality	Unequal exposure to the infectious environment
		Role ambiguity between doctors and nurses
	Professional ethics	Insufficient response to urgency requirements of the situation
		Low sense of responsibility in nursing services
	Job competency	Lack of knowledge and skills
		Inability in psychological adjustment and stress resistance
Coping styles	Active control and planning	
	Seeking support	
	Catharsis and staying focused	
Impacts on career	Specialized nursing skills	
	Scientific research ability	
	Management skills	

### Theme 1: Ethical challenges

The findings demonstrated four main themes: patients, inequality, professional ethics, and job competency.

### Category 1: People with COVID-19

According to the data, this theme included the subthemes of neglected patient rights and the lack of emotional support.

#### Subcategory 1: Neglected patient rights

Most of the interviewees mentioned a sharp increase in cases in a short period of time due to the sudden outbreak of COVID-19. In the early days, with the limited medical resources in Wuhan, patient rights such as the rights to choose treatment plans, the rights to know, and the rights of personal security were oftentimes neglected. Many critically ill patients were unable to effectively communicate, which led to the inability to choose treatment plans. At the same time, some patients did not have adequate safety precautions:

Some of the critical patients were not able to communicate, so we could not explain treatment plans to them. They could only accept what we offered. (N2)

In order to prevent the patients from being too anxious to worsen their conditions, I sometimes hid the truth from my patients, which made me very upset. (N6)

The protective mask used by the patient could not be replaced in time. (N11)

#### Subcategory 2: The lack of emotional support

As gatherings could potentially increase the risk of contracting COVID-19, family members were not allowed in the ward, so the patients often felt lonely. Some patients lost their beloved family members due to COVID-19, resulting in a lack of family relationships and a negative attitude toward treatment. In order to decrease the prevalence of infection, nurses and patients need to maintian a certain distance when they communicate with each other, giving patients a sense of lack of security. Seeing this pain in their patients caused moral distress for nurses, who felt unable to provide patients with necessary support:

There was a patient who refused to cooperate and ate little, because of the loss of his family. (N1)

I saw the panic and fear in their eyes when I was keeping a distance from them. (N12)

### Category 2: Inequality

During the process of nursing COVID-19 patients, inequality was often experienced by nurses, which mainly included unequal exposure to the infectious environment and role ambiguity between doctors and nurses.

#### Subcategory 1: Unequal exposure to the infectious environment

The findings showed that nurses spent more time exposing themselves to a contagious environment because they had to check on their patients, set up infusion therapy, turn bed-ridden patients, and feed patients who could not take care of themselves. By contrast, doctors were exposed to the virus for a much shorter time:

We frequently needed to deal with patients, which increased the chance of infection, but doctors spent much less time in the ward. (N4)

#### Subcategory 2: Role ambiguity between doctors and nurses

Due to the differences in professional background, knowledge, and work expertise, different roles were formed between doctors and nurses.^20^ Nurses expected doctors to be proficient and responsible, to support, and to respect nurses and their work. However, interviewees mentioned that some doctors expected nurses to assume some of the doctors’ responsibilities, such as checking on patients’ condition by pulmonary auscultation and bedside blood gas analysis, making nurses feel unequal in status and that their role was not respected:

Doctors should examine and observe the patients’ condition personally, but in order to reduce the risk of infection, some of them asked nurses to view the patients’ monitoring parameters through video instead of entering the ward. (N6)

Some jobs we did should have been finished by the doctors. Some doctors even asked us to do it through phone calls. (N9)

### Category 3: Professional ethics

Ethical challenges concerning professional ethics mainly arise from insufficient response to urgency requirements of the situation and low sense of responsibility in nursing services.

#### Subcategory 1: Insufficient response to urgency requirements of the situation

Because of the contagious nature of COVID-19, medical staff need sufficient protective gear, such as splash-proof respirators, when intubating the trachea. They also tended to reduce the frequency of exposing themselves to an infectious environment in order to lower the chance of infection. These self-protection measures, however, sometimes took more time and led to failures in fulfilling ethical obligations:

There was a patient who needed endotracheal intubation, but the anesthetist took a long time to find the respirator before intubating. He paid too much attention to his self-protection.(N7)

#### Subcategory 2: Low sense of responsibility in nursing services

Interviewees mentioned that some medical staff worked slowly while rescuing patients and reduced operations to avoid aerosols. Reduced frequency of nursing activities could give patients more time to rest, but it might also reflect low sense of responsibility. Responses were often slow and reduced in frequency, and nurses found this upsetting as they felt patients were not getting the best care:

Some nurses were worried about being infected, so they secretly reduced the frequency of helping patients turn over and rubbing their backs. (N3)

Time is precious in saving lives. A patient suddenly fainted, but my colleague took a long time walking towards him before eventually giving a hand. (N15)

### Category 4: Job competency

This theme included two subthemes, which were lack of knowledge and skills and inability in psychological adjustment and stress resistance.

#### Subcategory 1: Lack of knowledge and skills

Nurses had a lack of knowledge and skills as they needed to adapt to the new work environment and job responsibilities, and complete the role change in time. Due to the limited experience in treating infectious diseases, some faced challenges in organizational skills and treatment capacity. Because of the psychological fragility of COVID-19 patients, special psychological nursing measures were needed. Nurses should help patients overcome the fear and anxiety caused by infectious diseases, which put pressure on nurses concerning psychological nursing skills:

I used to do surgical nursing, but now internal medicine knowledge is needed for nursing COVID-19 patients, and I am not familiar with that. (N8)

Patients were in pain, but I didn’t know how to comfort them. (N13)

#### Subcategory 2: Inability in psychological adjustment and stress resistance

COVID-19 is a public health event drawing much attention from society and places heavy pressure on nurses. For patients with underlying diseases, COVID-19 infection advances more quickly than usual. As a result, nurses were constantly worrying about their inability to treat patients, their own safety and that of their colleagues, and the possibility of having to compromise their health. They felt powerless in psychological regulation and stress resistance:

An elderly patient was suffering from wheezing, and it became increasingly severe. None of the treatments could ease her symptoms. She said “help” to me trembling, and I burst into tears. (N5)

I was worried that I was not doing my job well, this kept me from falling asleep. (N18)

### Theme 2: Coping styles

Research findings demonstrated three main themes: active control and planning, seeking support as well as catharsis, and staying focused.

### Category 1: Active control and planning

Through active learning, the nurses established prone position ventilation on continuous renal replacement therapy (CRRT), and other nursing groups developed appropriate nursing plans for COVID-19 patients and held regular case-based seminars so as to contain the risk of infection, increase the recovery rate of patients, give better care to them, and improve self-confidence and sense of accomplishment in clinical practice:

Through learning, I acquired medical nursing skill, especially for COVID-19 patients in a short time. (N2)

Since the implementation of group nursing, we were collectively discussing special cases every day and our job became much easier. (N14)

As the Diagnosis and Treatment Protocol for COVID-19 is constantly updated, I have also been studying all the time. (N17)

### Category 2: Seeking support

By seeking help, nurses have received support from the state, society, and hospitals. The state offered policy support to nurses, including higher salaries, career development, and giving honorary title, to give them a better sense of professional recognition. Society funded more medical protective supplies. Hospitals provided support from management systems, professional knowledge training, and other aspects in order to help nurses better manage COVID-19, regulate nursing behavior, and coordinate their cooperation with doctors:

Hospitals have provided more masks for patients and have asked them to change regularly. (N10)

We reported the responsibility issues of doctors and nurses to the ward coordinators. They helped define division of labor between doctors and nurses. (N11)

My work unit gave a lot of care to my family, so I can work in Wuhan to treat COVID-19 patients without any concerns. (N18)

### Category 3: Catharsis and staying focused

The interviewees mentioned that they had been well aware that excessive occupational pressure and negative emotion would affect the process of nursing as well as their physical and mental health. They found catharsis and staying focused on their jobs helpful when it came to overcoming these negative influences. Nurses acquired emotional comfort by focusing on nursing jobs, or increasing communications with their colleagues, or making more contacts with their family members and friends. They also tried developing some hobbies in their spare time, through which they could release their pressure and anxiety accumulated during the process of nursing. These methods helped them restore their energy and also proved effective in controlling or even eliminating negative emotions:

One day, I felt that the working pressure was too high to bear, so I burst into tears hiding in bathroom, and I became relaxed after crying. (N1)

Every time I made a video call with my family, I would calm down quite a bit. (N3)

I tried to stay focused by learning a song, and music gave me strength. (N9)

### Theme 3: Impacts on career

The results were manifested in three subjects: specialized nursing skills, scientific research ability, and management skills.

### Category 1: Specialized nursing skills

To cope with the ethical challenges brought on by nursing COVID-19 patients, nurses should gain knowledge and nursing skills on COVID-19, which could improve their clinical practice ability on infectious diseases. Its coping process also required nurses to build nursing strategies with critical thinking and choose effective modes so that their clinical decision-making ability would be enhanced. Dealing with ethical challenges also gives the nursing staff better ability of autonomous learning, cooperation, and coping:

I have improved my skills in nursing infectious patients, and I have also learned how to communicate with patients in a more effective way. (N2)

I know more about how to cooperate with my colleagues. (N7)

I now have a better idea of how to cope with public health emergencies. (N16)

### Category 2: Scientific research ability

As a new type of viral pneumonia, COVID-19 provides new research insights and opportunities to come up with new ideas. Nursing practices could help collect case data and form scientific questions. To cope with the ethical challenges, nurses should improve the ability of solving problems. Also, research findings of COVID-19 may be effectively applied to nursing practice, which could verify the research problems by clinical results:

I wrote a paper based on the management experience of ventilation therapy for COVID-19 patients. (N5)

Problems met during the process of nursing help to study the mechanism, precaution, and treatment of COVID-19. (N7)

### Category 3: Management skills

Through formulating nursing plans and participating in nursing COVID-19 patients, nurses have gained experiences in making plans, directing medical operations, coordination, and organization, which could improve the management skills in their clinical practice:

I will more effectively organize nursing jobs. (N1)

These experiences will help me better coordinate and organize nursing activities when I return to my unit. (N13)

## Discussion

Nurses worked in a new environment to help COVID-19 patients. They stood on the frontline and fully devoted themselves to disease control regardless of any danger. However, at the same time, they had to cope with the ethical challenges brought by COVID-19.

### Nurses faced ethical challenges on multiple fronts

The study results showed that the major ethical challenges encountered by nurses came from patients, inequality, professional ethics, and job competency. This result shares some similarities with Naseri-Salahshour and Sajadi’s^20^ research, which may be attributed to the nature of nursing work itself.

In this study, one theme of ethical challenges is neglected—patient rights. Some patients could not choose their treatment plans because they were too sick to speak. This is different from the case where patients are not allowed to participate in making their treatment plan.^21^ Both cases resulted in the patients not being able to choose their treatment plans, but in the former case, this situation was caused by patients’ critical condition, whereas in the latter case, patients were deprived of their rights to make a decision. COVID-19 affects entire families.^22^ Patients may lose their close ones because of COVID-19, and this loss could have a huge psychological impact on them, making them pessimistic. Nurses felt unable to provide patients with necessary support to comfort them, which caused moral distress for nurses. Another theme of ethical challenges is inequality encountered by nurses, involving the role ambiguity of the medical staff. Role ambiguity refers to the unclear definition of jobs, behavior expectations, responsibilities, and affiliation.^23^ Studies have shown that the difference in role status and cooperation problems between doctors and nurses are the major challenges for nurses in clinical practice.^24^ Nurses expect that doctors ought to perform their role well. However, when nurses’ expectation is not fulfilled or satisfied, they may feel they are not respected and hence generate an emotional backlash.

The ethical challenges concerning professional ethics are mainly manifested as insufficient response to urgency requirements of the situation, which might be related to the treatment and protection requirements of COVID-19. An infectious environment brings continuous occupational stress to nurses, which requires them to constantly psychologically adjust themselves. Such long-term adjustment can cause emotional exhaustion, and long-term mental pressure can easily bring about job burnout^25^ so that low responsibility is shown in clinical practice.

The ethical challenges caused by job competency are different from those arising from techniques in common clinical practice. To cope with the challenges posed by public health emergencies, nurses should receive professional training and only by it can they acquire nursing ability in previously unfamiliar fields.^26^ The COVID-19 set high standards for nurses’ psychological adjustment and stress resistance. Due to the urgency of fighting the COVID-19, the nurses in this study had relatively short training periods and lacked experience in nursing infectious diseases.^27^

### Positive coping styles of ethical challenges

Coping styles are individual behavioral or cognitive strategies for problem-solving.^28^ In the process of nursing COVID-19 patients, all nurses experienced different types and degrees of ethical challenges, but at the same time they adopted positive coping styles, mainly including active control and planning, seeking support, catharsis, and concentration, which also conformed to the research content of Zhang.^29^

Through active control and planning, nurses gave full play to the subjective initiative of individuals and teams, and thereby found ways to solve problems, achieved role adaptation, and reduced ethical conflicts and dilemmas. By obtaining social support, they felt respected and honored, and loved nursing career more, which increased nurses’ professional identity, and their initiative to abide by professional ethics and norms could also be enhanced.^30^ Through catharsis and concentration, nurses controlled their own unhealthy emotions; overcame excessive anxiety, fear, self-abasement, and other emotions; and maintained their mental health. Unhealthy emotions of nurses could result in job burnout, so the patients might be treated indifferently and the quality of nursing might be affected. In this study, nurses were aware of their unhealthy emotions and their possible negative effects. They took initiative to divert their attention, devoted themselves to the rescue work, and found suitable entertaining ways to deal with the ethical challenges posed by negative emotions. This coping process has improved the nurses’ understanding of life and professional values, converting negative challenges in the nursing process to positive and constructive challenges.

### Ethical challenges in nursing COVID-19 patients can contribute to career development

Career success refers to the positive psychological or work-related gains accumulated and acquired by individuals during their working experiences.^31^ Differences in professional development may be related to social experience challenges.^32^ The ethical challenges in nursing COVID-19 patients and their positive coping styles have promoted the nurses’ abilities of clinical practice, decision-making, self-learning, coordination, and cooperation, as well as psychological endurance. The experience of ethical challenges helped nurses to develop their professional responsibility and dedication and laid the foundation for their professional development. The challenges of pressures and psychological adjustment prompted them to quickly adapt to role changes, make psychological adjustments, and learn to accept and develop new roles.^33^ Such an ethical challenge experience enables nurses to better understand their own values and motivation for achievement, ascertain their ability and opportunity orientation, and establish a clear career path.

## Strengths and limitations

This study was conducted against the background of the outbreak of the new infectious disease COVID-19. It is the first study to examine the ethical challenges of nurses treating COVID-19. Second, results showed experiences to be multifaceted, including ethical challenges, relevant coping styles, and their impacts on careers. And the coping styles in this study could instruct nurses to cope with ethical challenges in clinical practice.

Due to the working environment and that interviewers and some interviewees adopted voice calls, the interviewees’ expressions and body language could not be observed. Thus, incomplete interview information may affect the credibility of the results. In addition, the interviewees are mainly from Shandong Province, so the research might have certain geographical limitations. If a wider group of participants can be mobilized, a more comprehensive understanding of the ethical challenges faced by the COVID-19 nursing group may be obtained.

## Conclusion

With the global threat posed by COVID-19, we studied the ethical challenges faced by nurses in caring for COVID-19 patients through in-depth one-on-one interviews. The topics of challenges include patients, inequality, professional ethics, and their working ability. Some moral challenges can result in job burnout and mental illness. Through interviews, it was found that the nurses responded positively to the ethical challenges they encountered, which may have a beneficial impact on their career development. It is recommended that the government ensure that adequate and appropriate personal protective equipment is provided to medical care staff working on the frontline, to ensure the safety of nurses and other health workers. Also, society and hospital administrators should give more respect, care, and understanding to COVID-19 nursing personnel and provide them with more humanistic care. It is recommended to set up care teams to provide early, full-course, personalized, and comprehensive psychological assistance to the nurses in order to prevent acute stress disorder (ASD) and post-traumatic stress disorder (PTSD).
